# Lymphoma Related to the Ventricular System: A Rare Case Report and Systematic Review of Intraventricular Lymphomas

**DOI:** 10.3390/medsci14020211

**Published:** 2026-04-24

**Authors:** Maksymilian Niemczyk, Justyna Fercho, Szymon Goldszmyt, Bogdan Jabłoński, Oskar G. Chasles, Jakub Soboń, Marcin Birski, Jacek Szypenbejl, Maciej Mielczarek, Marek Harat, Mariusz Siemiński, Jacek Furtak

**Affiliations:** 1 Scientific Circle of Neurotraumatology, Department of Emergency Medicine, Medical University of Gdańsk, 80-210 Gdańsk, Poland; szymon.goldszmyt@gumed.edu.pl (S.G.); bogdan.jablonski@gumed.edu.pl (B.J.); oskar.chasles@gumed.edu.pl (O.G.C.); jacek.szypenbejl@gumed.edu.pl (J.S.); mariusz.sieminski@gumed.edu.pl (M.S.); 2 Neurosurgery Department, 10th Military Research Hospital and PolyClinic SPZOZ in Bydgoszcz, 85-681 Bydgoszcz, Poland; drsobon@gmail.com (J.S.); marcin.birski@10wsk.mil.pl (M.B.); harat3156@10wsk.mil.pl (M.H.); jacek.furtak2019@gmail.com (J.F.); 3 Department of Emergency Medicine, Medical University of Gdańsk, 80-210 Gdańsk, Poland; 4 Lung Transplant Unit, Cardiac Surgery Department, Medical University of Gdańsk, 80-210 Gdańsk, Poland; 5 Neurosurgery Department, Stanisław Staszic Specialist Hospital, 64-920 Piła, Poland; maciejmielczarek87@gmail.com; 6 Faculty of Medicine, Bydgoszcz University of Science and Technology, 85-796 Bydgoszcz, Poland

**Keywords:** intraventricular lymphoma, intraventricular tumors, lymphoma, PCNSL, SCNSL, lateral ventricle, EBV infection, DLBCL, immunochemotherapy

## Abstract

**Background**: Intraventricular central nervous system (CNS) lymphoma is an atypical presentation of extranodal lymphoma, whether primary or secondary. The most commonly diagnosed subtype of lymphoma is diffuse large B-cell lymphoma (DLBCL). There is a documented relation of HIV, EBV and KSHV infections with lymphomagenesis. AIDS-related lymphomas (ARLs) are described as a defining illness of the acquired immunodeficiency syndrome (AIDS). This study presents a novel case and systematic review of clinical, radiographic and histopathological features of intraventricular lymphomas. **Methods**: We report on a 27-year-old woman with a left lateral ventricle DLBCL with surrounding edema treated with steroids. A systematic review of 147 additional cases (1977–2025) was conducted, analyzing patient demographics, tumor characteristics, clinical features, imaging, treatment, and outcomes. The tumor locations were divided into three groups depending on the extent of ventricular involvement. Descriptive statistics summarized findings. **Findings**: 147 cases (mean age, 54.2 years; range, 3–87; 63.3% male) were analyzed. Immunodeficiency in patients was unusual (6.1%). Fully intraventricular lesions were the most common presentation (52.4%), with systemic involvement solely in 10 cases (6.8%). The lesions were predominantly located in the lateral ventricles or fourth ventricles (46 times each), and bilateral involvement was noted 37 additional times. DLBCL was diagnosed in 101 cases (78.9%). **Interpretation**: Intraventricular involvement in central nervous system lymphoma poses a diagnostic and therapeutic challenge due to non-specific symptoms and atypical locations. Adding to the diagnostic difficulty of intraventricular masses in young patients, we wish to highlight that immunocompromised patients are a notably insignificant subgroup of patients in our study.

## 1. Introduction

Lymphoma of the central nervous system (CNS) is a rare manifestation of lymphoma involvement, broadly classified into two groups. Primary central nervous system lymphoma (PCNSL) is diagnosed when the tumor is restricted to the CNS at the moment of the initial diagnosis, while secondary central nervous system lymphoma (SCNSL) arises as a consequence of systemic disease. The most commonly diagnosed lymphoma subtype is diffuse large B-cell lymphoma (DLBCL). This subtype constitutes approximately 90% of PCNSL cases [1].

PCNSL accounts for 3% of all CNS tumors with an annual incidence rate of 0.47 cases per 100,000 people in the general population [2]. These tumors typically present with rather non-specific symptoms; only 50–70% of patients display focal neurologic deficits, and 40–50% of them, a similarly high proportion, show general cognitive and behavioral changes [3].

SCNSL, however, is likely to occur at a median of 5–12 months following the diagnosis of systemic non-Hodgkin lymphoma (NHL) [4]. Additionally, the majority of SCNSLs manifest as leptomeningeal disease, and the prognosis of CNS involvement is poor due to a median survival following the diagnosis of 2–6.5 months [4].

Generally, the frequency of lymphoma cases is documented as highly related to immunodeficiency. NHL has a 23 times higher incidence in patients with HIV compared to the general population, with about 7% of NHL cases being those with concurrent HIV infections [5]. PCNSL is thought of as a distinct indicator of the AIDS disease, and, moreover, PCNSL is occasionally considered as the first manifestation in the course of the disease (sudden onset of AIDS) [5]. Within the group of AIDS patients specifically, DLBCL is also the most frequent subtype, also described as the most common type of AIDS-related lymphoma (ARL) [5,6]. The role of Epstein–Barr virus (EBV) in lymphomagenesis is also of note. EBV is connected to a wide scope of B-cell lymphoproliferative disorders. Interestingly, EBV is implicated in the pathogenesis of many lymphoma subtypes, including Hodgkin lymphoma (HL) and Burkitt’s lymphoma (BL) [7]. Most lymphomas occurring in the AIDS setting seem to be additionally linked to oncogenic viruses such as Kaposi sarcoma herpesvirus (KSHV) and EBV [8].

Involvement of the ventricular system by lymphomatous disease is highly uncommon and unique. The most common location of CNS lymphoma is within the periventricular white matter, specifically the frontal lobes, basal ganglia, thalamus, posterior fossa, and corpus callosum [9,10]. Notably, the vast majority of lymphoma tumors are in contact with the cerebrospinal fluid (CSF) [9]. A comprehensive review of lymphomas presenting within the ventricular system was carried out, including cases of partial ventricular components in a multifocal presentation or isolated intraventricular tumors. The literature review identified 147 cases of such nature.

This report presents a novel case of a 27-year-old woman presenting with diffuse large B-cell lymphoma in the left lateral ventricle, adding to the current, limited body of knowledge regarding this matter. This case highlights the diagnostic complexities of intraventricular masses in young patients.

## 2. Case Report

### 2.1. History and Examination

A 27-year-old right-handed woman was admitted to our center for surgical treatment of a previously diagnosed tumor in the left lateral ventricle.

The patient’s medical history included several months of memory and concentration difficulties. At the end of May 2025, she developed frontal headaches, motor aphasia, reading difficulties, and right temporal hemianopia.

Due to these symptoms, she was hospitalized in a neurology department from 29 May to 13 June 2025. Diagnostic workup included contrast-enhanced head MRI, which revealed a lesion in the left lateral ventricle accompanied by finger-like edema of the left cerebral hemisphere and smaller lesions in the posterior third ventricle. Corticosteroid therapy was initiated, resulting in a complete resolution of visual disturbances. At discharge, neurological examination revealed no significant deficits. At this point, the preliminary diagnosis indicated a low-grade proliferative process, and, therefore, a lumbar puncture was not performed.

The patient was a non-smoker with no history of epilepsy, any comorbidities, or allergies. Her Karnofsky Performance Status (KPS) score was 90.

A follow-up MRI performed on 2 July 2025 demonstrated regression of the left lateral ventricle lesion. Consequently, the planned stereotactic biopsy was canceled, and the patient was discharged home.

Serological screening was negative for human immunodeficiency virus (HIV) antibodies and Toxoplasma gondii antibodies. Epstein–Barr virus (EBV) serology demonstrated an IgM level of 26 U/mL and an IgG level of 78 U/mL, indicative of an EBV infection and consistent with an acute or recent primary infection. Hepatitis B virus (HBV) surface antigen (HBsAg) was non-reactive.

Tests such as double/triple-hit FISH, MYC IHC, MUM1/IRF4, Hans algorithm, EBV EBER ISH, MYD88, and CD79B mutations were not available at our center.

Two months later, she presented with right-sided facial and headache pain (intermittently also left-sided) and redness of the left eye. She reported an episode of sinusitis one week earlier.

On 24 September 2025, a stereotactic biopsy of the lesion was performed. The intraoperative preliminary diagnosis suggested a low-grade proliferative process. Subsequent histopathological examination confirmed diffuse large B-cell lymphoma. The patient remained in good general condition after the procedure. Postoperative CT showed no abnormalities, and she was discharged in good condition.

On 28 November 2025, a PET scan was performed. Additional small lesions were found surrounding the main hyperdense lateral ventricle lesion, with a similar lesion in the cerebellar vermis. These results correspond to a highly aggressive multifocal proliferation process within the central nervous system. No other abnormalities were found outside of those described.

The patient received immunochemotherapy following the R-MTX-AraC regimen (rituximab, methotrexate, and cytosine arabinoside). Three cycles were administered between October 2025 and December 2025, after which the immunochemotherapeutics were withdrawn due to worsening of the patient’s general condition and the development of a headache, aphasia, and confusion. An emergency MRI imaging showed progression of edema, later treated with dexamethasone and mannitol.

Upon follow-up with the primary hematologist during the writing of this current report, it was confirmed that the clinical course subsequently led to the patient’s passing.

### 2.2. Histopathological Findings

Pathological examination of the lateral ventricle mass revealed scant tissue fragments of neoplastic origin, initially suggesting astrocytic differentiation. The specimen showed low-to-moderate cellular density with infiltration by small lymphocytic cells in a glial background. Immunohistochemistry demonstrated a very high Ki-67 proliferation index (100%). Tumor cells were positive for CD20, CD79a, Bcl-2, and Bcl-6, and negative for GFAP, pan-cytokeratin (CK PAN), and CD3.

### 2.3. Operative Protocol

**Figure 1:**
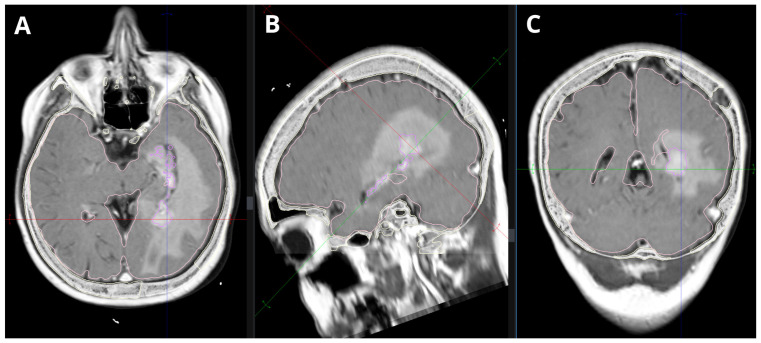
MPR merge CT + post contrast T1-weighted MRI showing an intraventricular homogeneously contrasted tumor inside the temporal horn of the left lateral ventricle. The tumor is growing along the choroid plexus. (**A**) Axial plane. (**B**) Saggital plane. (**C**) Coronal plane.

**Figure 2:**
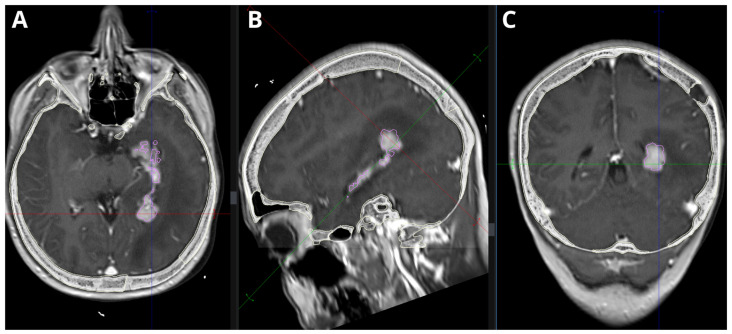
MPR merge CT + FLAIR-weighted MRI showing an intraventricular tumor with extensive edema of the surrounding temporal lobe structures. (**A**) Axial plane. (**B**) Saggital plane. (**C**) Coronal plane.

**Figure 3:**
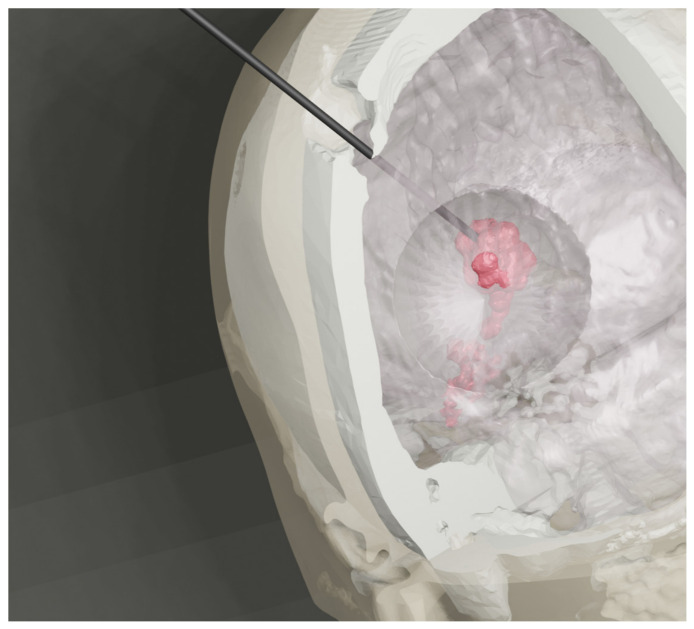
Preoperative 3D reconstruction of a diffuse large B-cell lymphoma in the lateral ventricle. The lesion is marked in red for visual reference, demonstrating its volume and spatial relationship to the ventricular system and surrounding structures. The image also illustrates the biopsy trajectory.

Under local anesthesia, a stereotactic frame was applied to the patient’s head, followed by a CT scan. The CT images were fused with prior MRI scans to plan the biopsy trajectory (Figure 1 and Figure 2). Our report includes a 3D preoperative reconstruction of the lesion (Figure 3).

The patient was then transferred to the operating room, where the frame was fixed. A skin incision was made over the entry point, and a burr hole was created in the left parietal region.

A biopsy needle was inserted along the planned trajectory, and tissue samples were obtained for histopathological examination. Intraoperative smear examination suggested a low-grade proliferative process (favoring low-grade glioma). The burr hole was sealed with bone wax, the wound was closed in layers, and a sterile dressing was applied.

Postoperatively, the patient’s neurological status remained unchanged.

## 3. Methods

### 3.1. Search Strategy and Protocol

**Figure 4:**
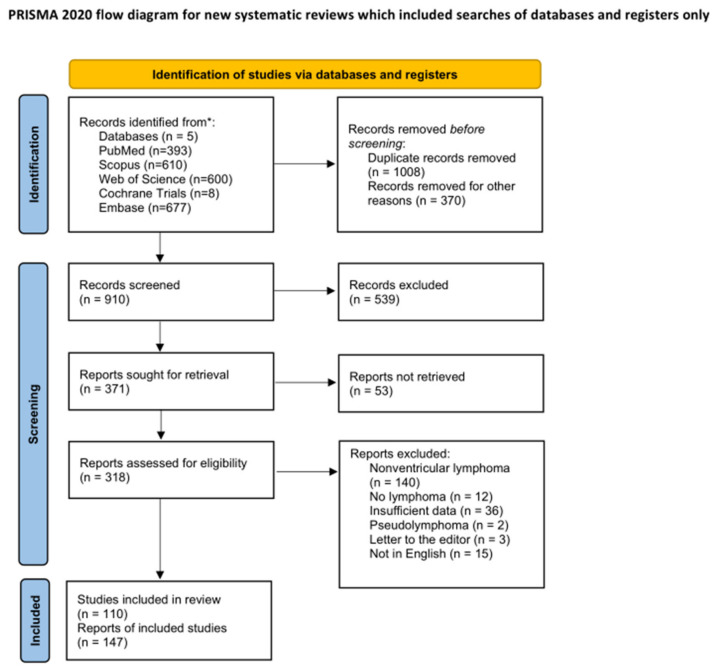
PRISMA Flowchart.

This systematic review was conducted and reported in accordance with the Preferred Reporting Items for Systematic Reviews and Meta-Analyses (PRISMA) 2020 statement [11] (Figure 4).

A comprehensive search strategy was developed using general keywords related to intraventricular lymphomas, with the aim of identifying all relevant studies reporting cases of adequate quality. Multiple preliminary combinations of terms and filters were tested to optimize the sensitivity and specificity. The final search query consisted of the following terms, combined with Boolean operators: lymphoma and (“lateral ventricle” or “third ventricle” or “fourth ventricle” or “ventricular system” or intraventricular or “ventricular lesion” or “ventricular tumor” or “cerebral ventricles”).

No language, date, or other filters or restrictions were applied.

The search was executed across five major scientific databases: PubMed, Scopus, Web of Science, Cochrane Central Register of Controlled Trials (Cochrane Trials), and Embase. This yielded a total of 2288 records. A custom web application developed by one of the authors (synthos.app) was used to combine search results from different scientific databases (PubMed, Scopus, Embase, and Web of Science) into one set of papers that was subjected to abstract screening. The program automatically removed duplicate entries and counted that 1008 articles were removed. As a result, 910 unique records were identified and advanced to the title/abstract screening phase. The program combines search results downloaded from the scientific databases and, by using the DOI identifier as a key value, finds papers that exist in more than one database. If a paper has more than one instance in the combined dataset, each entry except the first one is counted as a duplicate. All entries with the same key value (DOI) are also compared to each other to unify the papers’ metadata (title, abstract, authors, keywords, PMID, direct URL).

This systematic review was registered and accepted in the PROSPERO database. (registration number: CRD420251127227, available at https://www.crd.york.ac.uk/PROSPERO/view/CRD420251127227 (accessed on 21 April 2026).

### 3.2. Selection Criteria

Inclusion and exclusion criteria were predefined through consensus among multiple authors and finalized by the most senior investigators. The inclusion criteria encompassed peer-reviewed case reports and case series describing patients with intraventricular lymphoma (whether primary or secondary involvement of the ventricular system), irrespective of the size or extent of the neoplastic ventricular component, and required diagnosis to be confirmed by a histopathological examination of the tumor tissue obtained either intraoperatively (via biopsy or resection) or at autopsy. The exclusion criteria included studies that were not published in English, publications lacking essential metadata, studies for which the full text could not be retrieved, and cases involving multiple concurrent central nervous system tumor diagnoses (to ensure diagnostic specificity). The screening process occurred in two sequential stages, consisting of an independent title and abstract screening, followed by a full-text assessment of potentially eligible records. Both stages were conducted manually and independently by two reviewers, with any discrepancies resolved through discussion and senior authors making the final decision when a consensus could not be reached. The data from all included studies were extracted to enable subsequent statistical analysis.

### 3.3. Data Extraction and Statistical Analysis

The screening process identified 110 eligible studies that collectively described 147 cases of intraventricular lymphoma. From each included publication, relevant data points were systematically extracted. These encompassed study-level characteristics (e.g., year of publication and study design), patient demographic information (e.g., sex and age at diagnosis), and clinical details related to presentation, diagnostic workup, histopathological findings, treatment approaches, and outcomes (where reported). All extracted data were compiled into a structured spreadsheet.

Statistical analysis included both descriptive and inferential methods. Categorical variables were summarized as frequencies and percentages. Continuous variables were reported as means ± standard deviations (SD), medians, minimum and maximum values, and ranges. The proportion of missing data was calculated and expressed as a percentage of the total study population for each variable of interest.

The frequency distribution of age was visualized using a histogram, with each bar representing one decade of life.

Inferential statistical tests were selected according to the type, distribution, and number of observations. All hypothesis tests were two-tailed, with the significance level (α) set at 0.05. Statistical analyses and graphical representations were performed using GraphPad Prism version 10.6.1 for Windows (GraphPad Software, Boston, Massachusetts, USA; www.graphpad.com).

## 4. Results

We analyzed 110 papers (1977–2025) containing 147 cases of lymphoma arising at least partially within the ventricular system [12,13,14,15,16,17,18,19,20,21,22,23,24,25,26,27,28,29,30,31,32,33,34,35,36,37,38,39,40,41,42,43,44,45,46,47,48,49,50,51,52,53,54,55,56,57,58,59,60,61,62,63,64,65,66,67,68,69,70,71,72,73,74,75,76,77,78,79,80,81,82,83,84,85,86,87,88,89,90,91,92,93,94,95,96,97,98,99,100,101,102,103,104,105,106,107,108,109,110,111,112,113,114,115,116,117,118,119]. The research team performed data extraction and analysis without artificial intelligence tools. The data was compared, summarized, and presented in Table 1. Variables included patient demographics, tumor characteristics, radiographic features, clinical symptoms, treatments, and outcomes. Descriptive statistics summarized findings, with missing data noted. Additionally, inferential tests were conducted in order to discover correlations between the data presented.

The patient, whose anonymous clinical history was presented in this report, provided informed consent for publication of her case.

**Figure 5:**
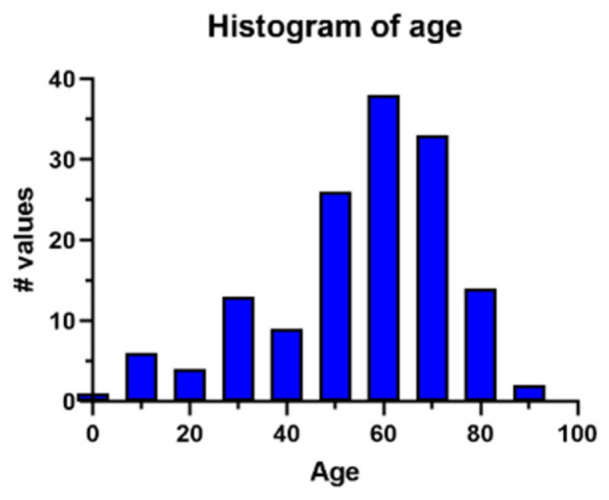
The age distribution.

Of the 147 patients, 54 (36.7%) were female, and 93 (63.3%) were male. The mean age at diagnosis was 54.2 years (range: 3–87). The mean age for males and females was 52.1 and 57.9 years, respectively. The distribution of patient age is presented in Figure 5.

The study population comprised patients aged 3 to 87 years with a standard deviation (SD) of 18.3. The median age was 59.

**Figure 6:**
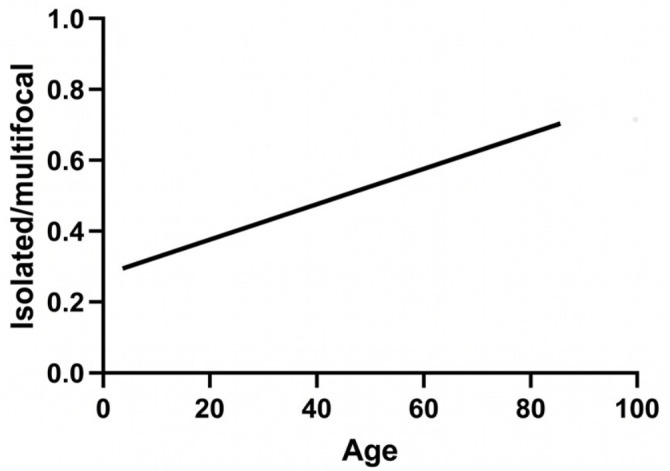
The simple logistic regression of age and lesion type.

Each additional year of life increased the odds of multifocal lesions by 2% (OR = 1.02, 95% CI: 1.003–1.041, *p* = 0.0235). The assumption of linearity between age and the logit of the outcome was assessed and confirmed via the Box–Tidwell transformation. This result is illustrated in Figure 6.

**Figure 7:**
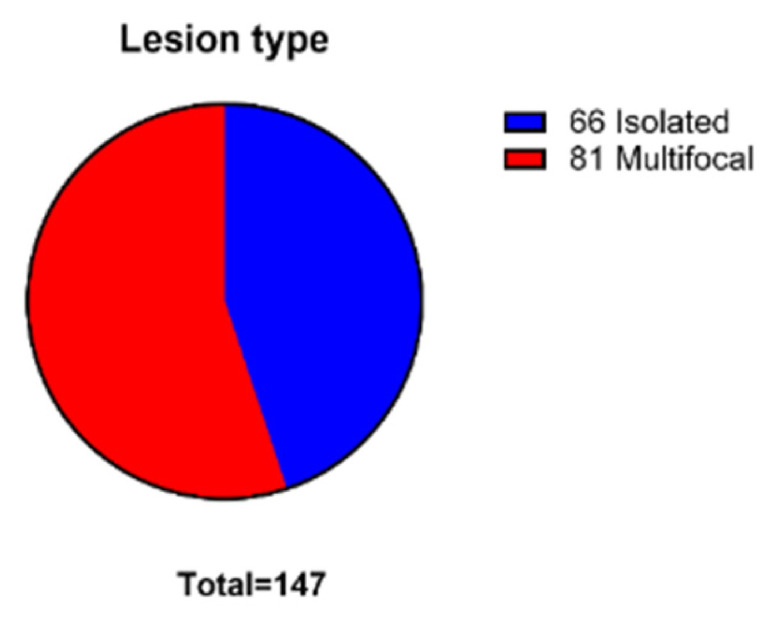
The lesion quantity per case distribution.

The tumor location was divided into groups based on several factors. Firstly, the lesions mostly presented in a multifocal manner 81 (55.1%) times, with 66 (44.9%) cases demonstrating a singular tumor, as seen in Figure 7.

Furthermore, lymphoma was solely systemic 10 (6.8%) times, and 137 (93.2%) more were confined within the central nervous system with no peripheral sightings.

**Figure 8:**
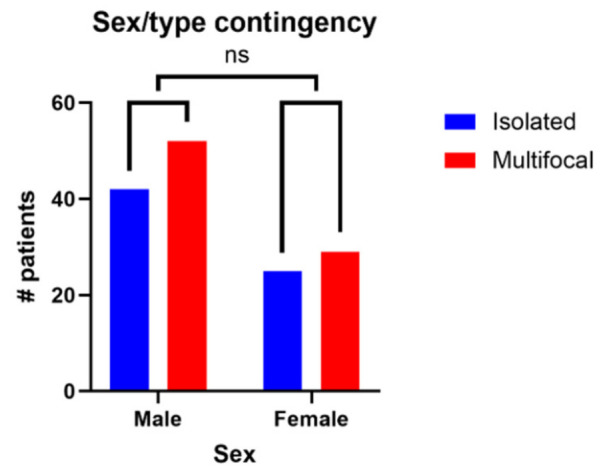
Sex/type contingency.

Regarding multifocality, the difference between the sexes was not significant (two-sided *p*-value = 0.8654, Fisher’s exact test). The sex/type contingency is shown in Figure 8.

Additionally, we introduced a categorization method comprising three subgroups based on the lymphoma’s relation to the ventricular system:

- Intraventricular (IV)—a singular lesion or a multifocal presentation contained within the ventricular system entirely;
- Ventricle-predominant (VP)—a singular lesion partially protruding into a ventricle, or multifocal lesions presenting intraventricularly no less than 50% of the time;
- Ventricular component (VC)—a multifocal intraventricular presentation consisting of less than 50% of tumors, which have arisen in the ventricular system; to be considered in the review, the VC lesion had to have been an element of the initial presentation.

The most common variant was an IV lymphoma, accounting for 77 (52.4%) cases, VP lymphoma showed 60 cases (40.8%), and VC lymphoma was the rarest subgroup with 10 (6.8%) patients.

Lateral ventricle involvement was the most frequent diagnosis, represented by 46 cases where a single lateral ventricle was occupied and an additional 37 times when the lymphoma was located bilaterally (43.9% in total). The lesions were occupying the fourth ventricle 46 times (24.3%) and the third ventricle 41 times (21.7%). Specific structures such as the septum pellucidum and corpus callosum were also involved, accounting for 10 (5.3%) and nine (4.8%) cases, respectively.

Immunodeficiency within this patient group was considerably rare. Only nine people were immunodeficient (6.1%), five of whom were linked to immunosuppressant intake (3.4%). The remaining three patients had concurrent neoplastic diseases, and one had a diagnosis of AIDS (0.7%). EBV was described in four patients. Furthermore, five patients developed a respiratory system infection; there was one more case of Acinetobacter baumannii and Pseudomonas each. One additional post-treatment infection in a patient was not otherwise specified.

We summarized the morphological and radiographic aspects of lymphomas in the patients along with symptoms collected up until the patients’ treatment. The presence or absence of these features was severely underreported, providing a fragmented understanding of the disease’s initial presentation.

- A hemorrhage was the most common characteristic, present 11 out of 17 times where its presence was reported (64.7%).
- Necrotic foci within the lymphoma lesion were present 10 out of 17 times where their presence was reported (58.8%).
- Midline shift was displayed in imaging five out of 10 times where its presence was reported (50%).
- Calcifications within the tumor were the least frequent presentation, with four out of 22 times where their presence was reported (18.1%).
- Hydrocephalus was identified on imaging in 26 out of all 147 cases (17.7%)

**Figure 9:**
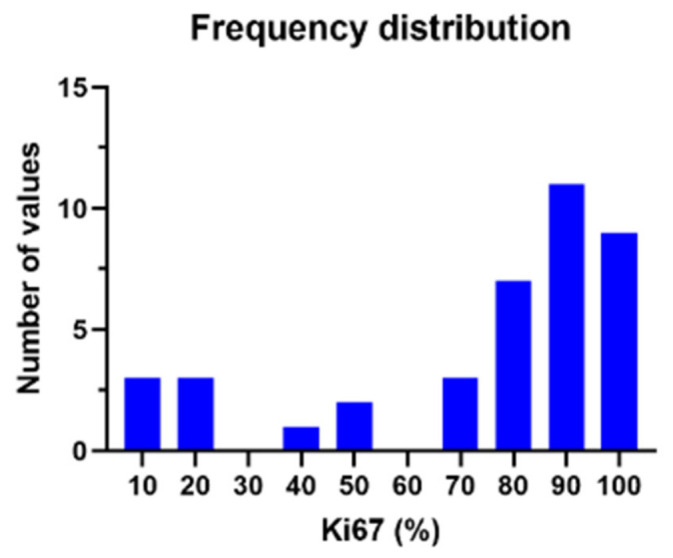
The Ki-67 proliferation index distribution.

The Ki-67 protein index was reported in 41 cases. The mean is 73.2% with a standard deviation of 30. The minimal Ki-67 value reported was 7%, and the value of 100% was noted in six patients. This distribution is illustrated in Figure 9.

Overall, 69 cases (68.3%) of diffuse large B-cell lymphoma did not have a specified subtype mentioned in their reports. Four out of 10 patients under 20 years old were diagnosed with diffuse large B-cell lymphoma. The specific distribution of histological subtypes is presented in Table 2.

Follow-up data for 97 patients were numerically defined. The mean follow-up is 15.75 months with a standard deviation of 21.3 (range: 0–124 months). We found that 30 patients out of 147 did not have any follow-up data, and 20 cases did not have specific numbers describing their follow-up period. We found that 15 of them were still alive at the time of publishing their case, four died at some point during the follow-up period, and one patient was deceased before treatment.

## 5. Discussion

Intraventricular involvement by central nervous system (CNS) lymphoma is an exceedingly rare entity, often overlooked due to its atypical location compared with the more common periventricular parenchymal presentation [9,10,33,117]. This study presents the largest systematic review to date of intraventricular lymphomas, encompassing 147 cases spanning nearly five decades (1977–2025), alongside a novel case of a diffuse large B-cell lymphoma (DLBCL) in the lateral ventricle, third ventricle, and cerebellar vermis of a young immunocompetent woman.

Our analysis reveals several key demographic and clinical features that distinguish intraventricular CNS lymphoma from the broader primary CNS lymphoma (PCNSL) population. The mean age at diagnosis was 54.2 years, with a notable male predominance (63.3%), consistent with prior reports of PCNSL epidemiology [1,2]. However, immunodeficiency—long considered a hallmark risk factor for CNS lymphoma—was strikingly uncommon in this cohort, affecting only 6.1% of patients, with HIV-related disease in just one case (0.7%). This contrasts sharply with traditional associations of PCNSL with human immunodeficiency virus (HIV), Epstein–Barr virus (EBV), and Kaposi sarcoma-associated herpesvirus (KSHV) infections, where AIDS-related lymphomas frequently manifest in the CNS and exhibit high EBV positivity (>90% in immunocompromised cases), multifocality, ring enhancement, necrosis, and hemorrhage [5,6,8]. The low prevalence of immunosuppression in our series suggests that intraventricular presentation may represent a biologically distinct subset, potentially driven by alternative pathogenetic mechanisms in immunocompetent hosts, as supported by emerging evidence of increasing PCNSL incidence in immunocompetent (particularly elderly) populations since the 1970s, while HIV-associated cases have declined with antiretroviral therapy [3,120].

Fully intraventricular lesions (IV group) constituted the majority (52.4%), followed by ventricle-predominant (VP, 40.8%) and ventricular-component (VC, 6.8%) cases. The lateral and fourth ventricles were equally affected (approximately 46 cases each), with bilateral lateral ventricle involvement adding further complexity in 37 instances. These findings underscore the predilection of lymphoma for ependymal and subependymal regions, likely related to proximity to cerebrospinal fluid pathways. Radiographically, hemorrhage (64.7%) and necrosis (58.8%) were frequent, while hydrocephalus occurred in only 17.7% of cases, lower than might be expected given the ventricular location.

The index case—a 27-year-old woman with a ventricular-predominant DLBCL—exemplifies several diagnostic challenges highlighted by our review. Initial non-specific symptoms (memory impairment, headaches, motor aphasia, and hemianopia) and dramatic steroid-induced regression delayed definitive diagnosis, a phenomenon well-described in CNS lymphoma due to apoptotic sensitivity to glucocorticoids [14,17]. The young age and absence of immunodeficiency further contributed to diagnostic difficulty, as clinicians may prioritize glioma or other primary brain tumors in this demographic. Ultimately, stereotactic biopsy confirmed high-proliferative DLBCL (Ki-67 100%, CD20+, CD79a+, Bcl-2+, Bcl-6+), aligning with the review’s finding that DLBCL comprises approximately 79% of intraventricular cases and exhibits high proliferative indices (mean Ki-67 of 73.2%).

Age emerged as a significant predictor of lesion multiplicity: each additional year increased the odds of multifocal disease by 2% (OR 1.02, 95% CI 1.003–1.041, *p* = 0.0235). This association may reflect cumulative molecular alterations or differences in immune surveillance with advancing age. Our finding that age is associated with multifocal disease must be interpreted with caution. Because this study relies on aggregated case reports, it is subject to publication bias; clinicians are more likely to publish cases that are clinically atypical or radiographically extensive. Furthermore, we cannot exclude the possibility of confounding by secondary CNS involvement (SCNSL), which is more prevalent in older populations with systemic non-Hodgkin lymphoma. In this aggregated sample, age was suggestively associated with a higher risk of multifocal disease, though this remains a hypothesis-generating finding requiring validation in prospective cohorts.

Systemic involvement was rare (6.8%), supporting the notion that most intraventricular lymphomas are primary CNS confined, potentially conferring a more favorable prognosis than secondary CNS involvement (median survival 2–6.5 months in historical series [4]).

Therapeutically, the intraventricular location poses unique challenges. Surgical resection is often limited by eloquent proximity and risk of hydrocephalus, rendering biopsy the primary diagnostic tool, as in our case. Standard PCNSL regimens (high-dose methotrexate, rituximab, consolidative radiotherapy) remain the cornerstone, though ventricular access may facilitate investigational intraventricular or intrathecal approaches. The outcome data were heterogeneous, with a mean follow-up of 15.75 months; longer-term prospective studies are needed to clarify the prognosis in this subgroup.

The proposed classification (IV, VP, and VC) offers a practical framework for stratifying ventricular involvement, which may guide surgical planning, radiation field design, and prognostic counseling. By including cases with even minor ventricular components, our review provides a comprehensive view previously lacking in the literature.

Comparisons with prior studies reinforce these observations. A 2019 literature review of ventricle-predominant PCNSLs noted DLBCL as less common (38% of diagnoses) than in broader PCNSL series, with multifocality, edema, and advanced age as defining features, and age as a predictor of multifocal disease with prognostic implications [33,120]. A 2010 cohort of 38 PCNSL patients (92% immunocompetent) reported common symptoms including cognitive dysfunction (58%), weakness (28%), headache (18%), and gait difficulty (18%), with seizures being the least frequent symptom (13%) and multifocal-to-solitary ratios of approximately 53% to 47%—patterns that are similar to our review—despite minimal ventricular involvement in that cohort (only three patients with associated ventricular spread) [33]. This resemblance suggests that immunocompetency primarily shapes clinical presentation, while localization influences additional features.

In immunocompetent patients, morphological and radiographic properties typically include hypo- or isointense periventricular lesions with perilesional edema, often as a single, solid, well-defined intra-axial mass; hemorrhage, necrosis, or calcifications are rarer and more suggestive of immunocompromise [9,121]. AIDS-related and HIV-negative DLBCL cases share morphological and genetic similarities but exhibit slight differences, with immunocompromised tumors more frequently EBV-positive and showing greater pleomorphism [8]. In this current case, the patient’s serological screening indicated a recent EBV infection, likely triggering the lymphomagenesis.

Ultimately, these findings necessitate a paradigm shift in the clinical approach to intraventricular masses. Lymphoma must be elevated on the differential diagnosis for atypical ventricular lesions—particularly those exhibiting hemorrhage or necrosis—regardless of the patient’s age or immune status. Widespread adoption of the proposed IV, VP, and VC classification framework will not only standardize neurooncological reporting and guide safe surgical planning but also serve as a foundational baseline for future prospective multicenter registries. As it is an entirely original classification, it requires prospective validation.

### Limitations of the Study

Several limitations must be acknowledged. Our case report lacked a cerebrospinal fluid analysis since lymphoma was not the main suspicion until the final diagnosis. Additionally, many tests, such as double/triple-hit FISH, MYC IHC, MUM1/IRF4, Hans algorithm, EBV EBER ISH, MYD88, and CD79B mutations, are not available at our center. The retrospective design and reliance on published cases introduce a selection and publication bias, favoring diagnostically confirmed or unusual presentations. The data incompleteness (e.g., follow-up missing in 20% of cases, and 68.3% cases of DLBCL not having a specific subtype mentioned) precluded robust survival or pathobiological analysis. Treatment regimens and therapeutic responses were too inconsistently reported across the included case reports to permit systematic tabulation or analysis. Only English-language reports were included, potentially excluding relevant cases. Finally, molecular data (e.g., MYD88, CD79B mutations, and EBV status) were sparsely reported, limiting insights into pathogenesis.

## 6. Conclusions

This report presents a rare case of a diffuse large B-cell lymphoma (DLBCL) within the left lateral ventricle, third ventricle, and cerebellar vermis in an immunocompetent 27-year-old woman, complemented by the largest systematic review of intraventricular primary central nervous system lymphomas (PCNSL), aggregating 147 cases reported from 1977 to 2025. These findings challenge the traditional view of PCNSL as a malignancy strongly linked to severe immunodeficiency, such as HIV infection and Epstein–Barr virus reactivation. In the intraventricular cohort, overt immunosuppression was uncommon (6.1%), with HIV documented in only one case (0.7%), suggesting that intraventricular PCNSL is a distinct subgroup with markedly reduced dependence on immunodeficiency or viral lymphomagenesis compared to classic parenchymal PCNSL. This subgroup typically affects middle-aged patients (mean 54.2 years), shows male predominance, frequently involves fully or predominantly intraventricular sites, and features high rates of hemorrhage, necrosis, and aggressive DLBCL histology. The index case illustrates diagnostic challenges in immunocompetent individuals, including non-specific symptoms, rapid steroid responsiveness, and delayed recognition due to young age and atypical location. Despite the limitations (retrospective design, publication bias, English-language restriction, and sparse molecular data), this work advances understanding of intraventricular PCNSL as a unique variant. Multimodal evaluation with advanced neuroimaging, molecular profiling, and histopathology is crucial for diagnosis and management. Prospective multicenter studies are needed to clarify pathogenesis, optimize therapy, and define outcomes in this rare subgroup.

## Figures and Tables

**Table 1:** Symptom distribution and percentage.

Clinical Finding	Amount (Total *n* = 561)	[%]
Motor disturbance	127	22.6
Gait disturbance	50	
Paresis/palsy	35	
Motor weakness	32	
Other	10	
Mentation disturbance	93	16.6
Consciousness disturbance	41	
General malaise	45	
Cognitive decline	19	
Headache	67	11.9
Visual disturbance	60	10.7
Nausea/vomiting	53	9.4
Dizziness	48	8.5
Memory disturbance	30	5.3
Altered sensorium	28	5.0
Speech disturbance	25	4.5
Fecal/urinary	11	2.1
Urinary incontinence	8	
Fecal incontinence	1	
Urinary retention	1	
Unspecified incontinence	1	
Seizures	10	1.8
Hearing disturbance	7	1.2
Abnormal/absent reflexes	5	0.9
Fever	2	0.35
Lymphadenopathy	1	0.17
Decreased libido	1	0.17
Endocranial hypertension	1	0.17
Dyspnea	1	0.17
Cough	1	0.17
Cauda equina syndrome	1	0.17
Neck stiffness	1	0.17
None (incidental finding)	1	0.17

**Table 2:** The distribution of histological subtypes of lymphoma.

Clinical Finding	Amount (Total *n* = 147)	[%]
DLBCL	101	78.9
GCB subtype	11	
Non-GCB subtype	14	
Double expressor	1	
Postgerminal center subtype	1	
High-grade	5	
Not further specified	69	
Burkitt’s lymphoma	8	6.3
Marginal zone/extranodal marginal/MALT	5	3.9
Small lymphocytic lymphoma	2	1.6
High grade B-cell NOS	4	3.1
B-cell, unspecified/ malignant B-cell NHL	4	3.1
Non-Hodgkin lymphoma NOS	4	3.1
Hodgkin’s lymphoma	2	1.6
Anaplastic large cel lymphoma, ALK-positive	1	0.8
T-cell lymphoma (non- anaplastic)	1	0.8
High-grade large T-cell lymphoma	1	0.8
Lymphoplasmacytic lymphoma	1	0.8
Intravascular lymphomatosis	1	0.8
Histiocytic lymphoma	1	0.8
Diffuse undifferentiated large cell lymphoma	1	0.8
Small cell lymphoma NOS	1	0.8
Not specified	3	0.23

## Data Availability

The original contributions presented in this study are included in the article/Supplementary Material. Further inquiries can be directed to the corresponding authors.

## Supplementary Materials

The following supporting information can be downloaded at https://www.mdpi.com/article/10.3390/medsci14020211/s1. PRISMA abstract checklist and PRISMA manuscript checklist.

## List of Abbreviations

AIDS	Acquired Immunodeficiency Syndrome
ARL	AIDS-Related Lymphoma
Bcl	B-cell Lymphoma (e.g., Bcl-2, Bcl-6)
BL	Burkitt’s Lymphoma
CD	Cluster of Differentiation (e.g., CD3, CD20, CD79a, CD79B)
CI	Confidence Interval
CK PAN	Pan-Cytokeratin
CNS	Central Nervous System
CSF	Cerebrospinal Fluid
CT	Computed Tomography
DLBCL	Diffuse Large B-Cell Lymphoma
EBER	Epstein–Barr Virus-Encoded Small RNAs
EBV	Epstein–Barr Virus
FISH	Fluorescence In Situ Hybridization
FLAIR	Fluid-Attenuated Inversion Recovery
GFAP	Glial Fibrillary Acidic Protein
HBsAg	Hepatitis B Surface Antigen
HBV	Hepatitis B Virus
HIV	Human Immunodeficiency Virus
HL	Hodgkin Lymphoma
IgG	Immunoglobulin G
IgM	Immunoglobulin M
IHC	Immunohistochemistry
IRF4	Interferon Regulatory Factor 4
ISH	In Situ Hybridization
IV	Intraventricular
KPS	Karnofsky Performance Status
KSHV	Kaposi Sarcoma Herpesvirus/Kaposi Sarcoma-associated Herpesvirus
MPR	Multiplanar Reconstruction
MRI	Magnetic Resonance Imaging
MUM1	Multiple Myeloma Oncogene 1
MYC/MYD88	Myelocytomatosis Oncogene/Myeloid Differentiation Primary Response 88
NHL	Non-Hodgkin Lymphoma
OR	Odds Ratio
PCNSL	Primary Central Nervous System Lymphoma
PET	Positron Emission Tomography
PRISMA	Preferred Reporting Items for Systematic Reviews and Meta-Analyses
R-MTX-AraC	Rituximab, Methotrexate, Cytosine Arabinoside
SCNSL	Secondary Central Nervous System Lymphoma
SD	Standard Deviation
VC	Ventricular Component
VP	Ventricle-Predominant
